# Research on psychosocial aspects of asthma in the Arab world: a literature review

**DOI:** 10.1186/s40248-015-0011-6

**Published:** 2015-04-15

**Authors:** Anas J Al-khateeb, Jamal M Al khateeb

**Affiliations:** Internal Medicine Department, Saint Michael’s Medical Center, 111 Central Avenue, Newark, New Jersey 07102 USA; Department of Counseling & Special Education, College of Education, University of Jordan, Queen Rania Street, Amman, 119942 Jordan

**Keywords:** Arab countries, Bronchial asthma, Knowledge, Psychology, Psychosocial aspects, Attitudes, Quality of life

## Abstract

The importance of psychosocial factors in the management of bronchial asthma has long been recognized. This paper offers a review of research published in the English language related to psychosocial aspects of bronchial asthma in Arab countries. Several databases (PubMed, Science Direct, Springer Link, ERIC, and PsychInfo) were searched using the following keywords: bronchial asthma, Arab countries, Algiers, Bahrain, Comoros, Djibouti, Egypt, Iraq, Jordan, Kuwait, Lebanon, Libya, Mauritania, Morocco, Oman, Palestine (West Bank, Gaza), Qatar, Saudi Arabia, Syria, Tunisia, Sudan, Somalia; United Arab Emirates, and Yemen. Thirty-two studies were conducted in 9 Arab countries. Almost all studies found were published in the last fourteen years with an apparent increasing rate in the last five years. In descending order, these studies addressed: knowledge of and attitudes toward asthma, quality of life, behavioral and emotional problems and factors related to academic achievement.

The main results of the studies reviewed were: (a) physicians’, school staff’s, and parents’ knowledge of and attitudes toward asthma were generally unsatisfactory, (b) in-service asthma education programs significantly impacted parent and staff knowledge and attitudes, and asthma management practices, (c) quality of life in children and adolescents was significantly adversely affected by asthma, (d) asthma was a common cause of school absenteeism, and had a significant negative impact on academic achievement of students, and (e) students with asthma had significantly higher rates of behavioral and emotional difficulties compared to students without asthma. The paper concludes with a discussion about the implications of these results and a call for further research in this area.

## Introduction

Bronchial asthma, a chronic condition affecting the lungs characterized by widespread, reversible, inflammation and narrowing of the airways [1], is one of the most common non-communicable chronic illnesses among children [2-4]. The incidence of asthma has been on the rise in the past four decades, especially in developed countries[5-7], with more than 300 million people affected worldwide [8].

## Review

It has long been thought that psychological factors may play an important role in bronchial asthma [9-13]. Research published during the last few decades illustrates that psychological disorders are more common in individuals having bronchial asthma, especially if it is poorly controlled [14,15]. For instance, children with asthma are more likely than children without asthma to have anxiety and depression [16-19].

Research over the past few decades also indicates that psychological factors may influence the symptoms and management of bronchial asthma in children and adults in many ways [12]. Further, research shows that psychological stress may worsen asthma symptoms [20]. Accordingly, the research literature emphasizes the importance of psychological interventions with children and adults having asthma [21]. Bronchial asthma is one of the leading causes of hospitalization and school absenteeism among school age children [2]. One study [22] reviewed and analyzed empirical studies which examined the relationship between psychosocial factors and asthma management and morbidity in the 1980s and 1990s. This study reported that evidence indicated that psychosocial factors can play an important role in symptom expression and management. According to the researcher, caregiver and child mental health attitudes and skills, and problem-solving skills pertaining to asthma management may be among the most important determinants of adherence and subsequent asthma morbidity. However, another study [23] compared psychosocial characteristics of children with asthma and children without asthma. Children’s behaviors were assessed by parents with 15 questions. The only significant difference between the two groups was in sleep patterns (children with asthma slept less well than children without asthma). The two groups did not differ significantly in all other items related to school/learning habits, level of activity, and communication/affection. The researchers concluded that psychosocial differences between children with asthma and children without asthma were less remarkable than expected.

A recent bibliometric study of bronchial asthma in Arab countries [24] revealed that before 2000, research on bronchial asthma in Arab countries was low. The Kingdom of Saudi Arabia ranked first among the Arab countries in asthma research, and the majority of this research has been published in English. This study, however, did not provide information on psychosocial aspects of asthma in the Arab region. Rather, it only estimated the contribution of each Arab country in asthma research per year, and grouped studies into ten very general categories (i.e., medicine, immunology and microbiology, pharmacology, biochemistry, and others). To the best knowledge of the authors, no other study reviewed and analyzed asthma research in Arab countries. Accordingly, a study investigating research on psychosocial aspects of asthma in Arab countries is justified.

Thus, the purpose of the present study was to review research on psychosocial aspects of bronchial asthma carried out in Arab countries.

Studies strictly addressing epidemiology, causes and risk-factors, and treatment of asthma were excluded from the current review. First, the methodology used in identifying and reviewing relevant studies is described. Second, the findings are presented and discussed, and implications for future research on the psychosocial aspects of bronchial asthma are offered.

## Methods

Our aim was to review and analyze research related to the psychosocial aspects of bronchial asthma in Arab countries published from 1990–2014 via. The search engines used were: PubMed, PsychINFO, ERIC, SpringerLink, and ScienceDirect. The keywords entered into the search engines were: bronchial asthma, Arab countries, Algiers, Bahrain, Comoros, Djibouti, Egypt, Emirates, Jordan, Iraq, Kuwait, Lebanon, Libya, Mauritania, Morocco, Oman, Palestine (Gaza and West Bank), Qatar, Saudi Arabia, Somalia, Sudan, Syria, Tunisia, and Yemen. Reference lists at the end of articles located were also viewed in order to find additional articles. The search was restricted to studies published in English language.

## Results

Table 1 shows author(s), country, sample, parameters, and main findings of each study. A total of 32 studies (26 full text articles and 6 abstracts) were found to be relevant for the purpose of this review. These studies were conducted in Egypt (8; 25%), Jordan (8; 25%), Saudi Arabia (7; 21.8%), Qatar (2; 6.3%), Tunisia (2; 6.3%), UAE (2; 6.3%), Bahrain (1; 3.1%), Kuwait (1; 3.1%), and Lebanon (1; 3.1%). The studies included a total of 71,765 participants (parents, physicians, teachers, and persons with asthma and without asthma of different ages).

**Table 1:** **An overview of studies on psychosocial aspects of** bronchial asthma **in Arab countries published in English from year 1990 to 2014**

Study		Sample	Parameters	Findings
Abdel Gawwad and El-Herishi 2007 [25]	Saudi Arabia	A random sample of 297 school staff (225 teachers, 50 administrators, and 22 social workers)	Knowledge of and attitudes toward asthma, and asthma management practices	An in-service training program involving pamphlets and demonstration of inhaler use significantly impacted staff knowledge, attitudes, and management practices.
Abdel Hai et al. 2010 [26]	Egypt	A total of 103 children and adolescents aged between 8 and 16 years	Reliability and validity of an Arab version of Juniper et al.’s Pediatric Asthma Quality of Life Questionnaire (PAQLQ)	The Arab version of the PAQLQ had high construct and discriminant validity as well as high reliability measured by internal consistency.
Abudahish and Bella 2006 [27]	Saudi Arabia	A random sample of 61 primary health care (PHC) physicians	PHC physicians’ knowledge of and attitudes toward asthma, and practices in asthma care	PHC physicians’ knowledge of and attitudes toward asthma were low. These physicians’ practices in asthma care were not satisfactory.
Al-Akour and Khader 2009 [28]	Jordan	A random sample of 326 parents of 7–17 year-old children with asthma	Quality of life (QoL) of parents having children with asthma	Parents’ self-reported QoL was moderate to positive. Parents experienced more limitations in the activities’ domain than in the emotions’ domain. Parents having older children, living in rural areas, and mothers of children with mild asthma reported higher levels of QoL.
Al-Akour and Khader 2008 [29]	Jordan	A random sample of 200 children (7–17 year-old) with bronchial asthma	QoL as measured by PAQLQ	Children’s QoL was very low, scoring more limitations in the domain of activities than in emotions and symptoms. QoL of younger, female children, living in rural areas were the lowest.
Al-Binali et al. 2010 [30]	Saudi Arabia	A convenience sample of 171 mothers of children with asthma	Mothers’ knowledge and behaviors concerning asthma using an Arab version of the Chicago Community Asthma Survey (CCAS)	Mothers lacked the necessary knowledge and practices to care for their children with asthma. Complications of bronchial asthma were the least known by mothers. Significant risk factors for poor knowledge and behaviors among mothers were mother’s illiteracy and young age and female sex of the child.
Albsoul-Younes et al. 2004 [31]	Jordan	A convenience sample of thirty children (ages 6–15 years) with bronchial asthma	Asthma control and QoL as measured by an adapted version of the “Living with Asthma Questionnaire” (LWAQ)	Improved QoL and better control of asthma were reported after the children switched from inhaled corticosteroids to a single inhaler device of Budesonide and/Formoterol.
Al-Dawood 2002 [32]	Saudi Arabia	Parents of 1482 schoolboys 141 (9.5%) of whom were diagnosed with bronchial asthma	Mean period of school absenteeism	Mean period of school absenteeism among schoolboys with bronchial asthma was 13.6 compared to 3.7 for classmates without bronchial asthma. School absenteeism among children with bronchial asthma was associated with younger age of children, lower socioeconomic status, history of pets at home, presence of a smoker at home, and hospital visit or admission.
Al-Ghamdy 2000 [33]	Saudi Arabia	A convenience sample of 606 children (under 13 years of age) with bronchial asthma	Socio-clinical profile and impact of bronchial asthma on children’s life style	Asthma’s adverse effects on children were: sleep problems, school absences, and relatively frequent hospitalization.
AlGewely et al. 2013 [34]	Egypt	A purposefully selected sample of 77 males and 63 females (ages 7–17 years) with bronchial asthma	Level of asthma control and QoL as measured by PAQLQ	QoL was significantly adversely affected by bronchial asthma. Determinants of QoL were: level of asthma control, use of systemic steroids, parental smoking, hospital admission, and difficulties in obtaining drugs.
Alnasir and Skerman 2004 [35]	Bahrain	A random sample of 1140 schoolteachers	Teachers’ knowledge about bronchial asthma and four other chronic illnesses using a questionnaire	Teachers’ knowledge of bronchial asthma and other chronic illnesses prevailing in local society was deficient. The researchers recommended the implementation of pre-service and in-service training programs for teachers regarding bronchial asthma and other chronic health problems.
Al-Nsour et al. 2013 [36]	Jordan	A random sample of 3,654 individuals aged 18 years or older selected from Behavioral Risk Factor Surveillance Survey of the Jordanian population	The association between mental distress, bronchial asthma and three other chronic conditions, and adverse health behaviors	People with bronchial asthma and other chronic health conditions were more likely to have mental distress than people without those health conditions. The researchers called for a concerted effort to strengthen mental health care services for individuals with bronchial asthma and other chronic diseases.
Al-sheyab et al. 2012(a) [37]	Jordan	A random sample of 261 students (ages 8–18) with bronchial asthma	Health-related QoL, self-efficacy to resist smoking, and knowledge of asthma self-management	The intervention implemented in this study (a peer-led education program) successfully educated their younger peers with bronchial asthma, with significant improvement in the three dependent measures in this study (QoL, self-management, and avoiding smoking).
Al-Shayeb et al. 2013 [38]	Jordan	A random sample of 72 grade 8–10 students having bronchial asthma who were smokers	Self-efficacy to resist smoking, asthma knowledge, and QoL	A peer-led asthma education program significantly improved adolescents’ self-efficacy to resist smoking, asthma knowledge, and all sub-domains of QoL.
Al-Sheyab et al. 2012(b) [39]	Jordan	A purposefully selected sample of 34 school girls aged 7–11 years with bronchial asthma	Feasibility and acceptability of an Arabic version of a peer-led asthma education program, and asthma knowledge and awareness	A peer-led asthma education program was found feasible and acceptable in a Jordanian school context. This program resulted in improved knowledge and awareness of asthma.
Al Zahrani et al. 2014 [40]	Saudi Arabia	A purposefully selected sample of 200 children and adolescents (aged 7–17 years) with bronchial asthma	QoL of children and adolescents with impaired asthma control and controlled asthma using the PAQLQ and Arabic version of the Asthma Control Test	Children and adolescents with controlled asthma had higher QoL scores compared to children and adolescents with impaired asthma control.
Bener et al. 1994 [41]	United Arab Emirates (UAE)	A convenience sample of 28,447 primary school children aged 6–14 years	School absenteeism resulting from bronchial asthma	The findings confirmed that asthma was a common chronic disease among primary school children in UAE (6.7%), and was a common cause of absenteeism from school (at least one day of school was missed by 62% of boys and 72% of girls).
Bener et al. 2007 [42]	Qatar	A convenience sample of 31,400 primary school children aged 6 to 12 years	School absenteeism resulting from bronchial asthma	10.4% children were diagnosed as having asthma and wheezing. Overall 8% of them were absent from the school for at least one day during the year. Most absenteeism occurred during spring and autumn for both boys and girls.
Benzarti et al. 2003 [43]	Tunisia	A convenience sample of 110 asthmatic patients aged from 18 to 70 years	QoL as measured by an Arabic version of “Chronic Respiratory Disease Questionnaire”	QoL was more affected among women than among men at the environment dimension. QoL was not influenced by age, length of disease or respondents’ educational attainment. Nevertheless, QoL was more affected when asthma was more severe and corticosteroids improved the overall QL.
Binsaeed et al. 2014 [44]	Saudi Arabia	A convenience sample of 158 children with asthma aged 4-11 years	Asthma control status as measured by the childhood asthma control test	Asthma control improved with the number of siblings, increased asthma knowledge of the caregiver (OR = 0.87, 95% CI = 0.81-0.93). Uncontrolled asthma increased with low household incomes and sharing a bedroom.
Elkishishy & Abu Hegazy 2010 [45]	Egypt	A convenience sample of 40 adolescents (aged 13–17 years) with asthma and 40 adolescents without asthma	Psychological stress, psychopathology and life satisfaction	Compared to adolescents without asthma; adolescents with asthma had more depression, psychopathology, distress and life dissatisfaction.
Fathy et al. 2014 [46]	Egypt	A random sample of 50 patients (mean age 28 years) with asthma and 50 patients without asthma (mean age 33 years)	Differences among patients with asthma and healthy controls in anxiety, depression and neuroticism	Both state and trait anxiety were found to be significantly higher in patients with asthma. However, no significant differences were found among participants with and without asthma in depression or neuroticism scores (hysteria; depression; obsession; and somatic, phobic and free-floating anxiety).
Hossny et al. 2009 [47]	Egypt	A purposefully selected sample of 422 children with bronchial asthma	Exacerbation of bronchial asthma	Psychological stress was among factors precipitating acute exacerbations of bronchial asthma
Kamal & Bener 2009 [48]	Qatar	A total of 699 school children who had repeated their grade in the academic years from 2003 to 2008	Factors associated with school failure	Asthma was the most common chronic disease among students who experienced school failure.
Kawafha & Tawalbeh 2014 [49]	Jordan	A multi-stage cluster sample of 74 schoolteachers randomly assigned to an experimental group and a control group	Knowledge about asthma as measured by the Asthma General Knowledge Questionnaire for Adults (AGKQA)	The asthma education program significantly improved the experimental group’s knowledge of asthma.
Mansour et al. 2014 [50]	Egypt	A convenience sample of 130 students with bronchial asthma (mean age of 14 years) and a random control group of students without bronchial asthma	Cognitive functioning (assessed through an Arabic version of the Revised Wechsler Intelligence scale for Children (WISC-R), the Auditory Vigilance Test, and the Figural Memory Test) and academic achievement (measured through mid-year test scores in of Arabic and mathematics subjects)	Bronchial asthma had a significant negative impact on academic achievement and cognitive functioning of students with bronchial asthma.
Panicker et al. 2008 [51]	Kuwait	A sample of 102 patients with asthma and a control group of 102 individuals without asthma aged 20–60 years	Psychological distress in patients with bronchial asthma as measured by the World Health Organization (WHO) - Five Well-Being Index	Patients with asthma were significantly more psychologically distressed than the control group. The level of psychological distress was significantly higher among younger, female patients with shorter duration of asthma.
Salama et al. 2010 [52]	Egypt	A simple random sample of 352 clinicians (general practitioners and pediatric specialists and consultants) engaged in direct childhood asthma care	Physicians’ knowledge, practices and attitudes toward and adherence to national and international asthma management guidelines assessed by a self-administered questionnaire	Physicians’ had poor knowledge and attitudes. About 25% of the studied physicians were not in agreement with the guidelines claiming that their disagreement was mainly due to patient factors (poor socioeconomic standards and poor patient compliance).
Samuel et al. 2011 [53]	Egypt	A convenience sample of 23 children with asthma and 23 children without asthma with an age range of 6–15 years	Intellectual functioning and psychosocial adjustment as measured by WISC-R and Pediatric Symptom Checklist (PSCL)	Cognitive abilities, psychosocial adjustment and academic achievement were negatively influenced by bronchial asthma.
Swadi 2001 [54]	UAE	A stratified sample of 81 students with bronchial asthma and 81 students without asthma with an age range of 6–13 years	Behavioral and emotional problems assessed by an Arabic version of Rutter Children Behavior Questionnaire (parents’ and teachers’ versions)	Students with asthma were reported to have significantly higher rates of behavioral and emotional difficulties compared to students without asthma.
Zaraket et al. 2011 [55]	Lebanon	A convenience sample of 389 parents of children having bronchial asthma aged 3 to 15 years	Parental perceptions and beliefs about childhood asthma and its management	Parents of children with asthma showed fear of social stigma and harbored considerable misperceptions about the disease. It was also found that asthma was an important cause for school absence.
Zendah et al. 2011 [56]	Tunisia	A convenience sample of 85 adult persons with asthma	QoL as measured by Juniper’s Quality of Life Scale	Factors that negatively affect QOL were: the intellectual level, asthma severity and the absence of treatment adjustment.

Only one study addressed the psychosocial aspects of bronchial asthma in Arab countries before 2000. Twenty (62.5%) of these studies involved non-probability samples. In descending order, the studies addressed the following topics: knowledge of and attitudes toward asthma, QoL, behavioral and emotional problems and factors related to academic achievement.

All studies used quantitative research methods, collecting data mainly through self-report questionnaires. The main results of the studies reviewed were as follows:

- Physicians’, school staff’s, and parents’ knowledge of and attitudes toward asthma were generally unsatisfactory.
- In-service asthma education programs significantly impacted parent and staff knowledge and attitudes, and asthma management practices.
- QoL in children and adolescents significantly adversely affected by asthma.
- Asthma was a common cause of school absenteeism, and had a significant negative impact on academic achievement of students.
- Students with asthma had significantly higher rates of behavioral and emotional difficulties compared to students without asthma. These difficulties included, but were not limited to: sleep problems, distress, depression, and anxiety.

## Discussion

The present study shows that research on psychosocial aspects of bronchial asthma in the Arab region is quite limited. Despite the increasing number of Arab studies examining psychosocial aspects of bronchial asthma in the Arab world, this type of research remains relatively limited. The average rate of research productivity in this area was about one study per year over the last quarter-century. Only 32 studies were conducted in the 22 countries comprising the Arab world over a period of 24 years. Likewise, only few psychological factors were addressed in these studies. Nonetheless, these studies shed light on some psychological factors that influence the quality of life, academic achievement, and behavioral and emotional development of children and adolescents with asthma in Arab countries.

One main result reported by the studies reviewed here was that physicians’, school staff’s, and parents’ knowledge of and attitudes toward asthma were generally unsatisfactory. Similar findings were reported by researchers in other countries. For instance, primary school teachers in Turkey lacked knowledge on triggers of asthma attacks and on the management of this disease [57]. In the USA, studies have revealed that lack of education for nurses, parents, and physicians were among major barriers to asthma management in schools [58]. In China, it was reported that the parents’ knowledge of asthma was generally poor [59].

It was also reported by studies reviewed that in-service asthma education programs significantly impacted knowledge and attitudes, and asthma management practices. This result is in agreement with those of many studies conducted in other countries showing that training courses could have a positive impact on asthma control [60] and that education programs pertaining to application of standard treatment guidelines improved asthma control [61].

Another main result reported by the studies reviewed was that quality of life in children and adolescents was significantly adversely affected by asthma. This finding is consistent with those of previous non-Arab studies which have shown that children having asthma generally tend to have lower levels of quality of life than children without asthma [62-64] and that quality of life decreased with increasing asthma severity [65]. Further, studies included in this review showed that asthma was a common cause of school absenteeism, and had a significant negative impact on academic achievement of students. Similar findings were reported in other countries [66,67]. Finally, a main finding in this review was that children and adolescents with asthma had significantly higher rates of behavioral and emotional difficulties compared to children and adolescents without asthma. Similar findings were reported by many researchers in other countries [68-70].

While published studies provided beneficial insights into psychological factors associated with bronchial asthma in Arab countries, much research remains to be conducted. Research in Arab countries has not yet addressed many important psychological issues related to bronchial asthma. For instance, very few studies analyzed in this review attempted to utilize psychological interventions (e.g., cognitive behavioral therapy, behavioral therapies, relaxation techniques) to increase the effectiveness of asthma management and treatment through procedures such as promoting adherence and asthma control, managing emotional triggers, adopting appropriate adjustment and coping styles, and improving knowledge and attitudes, among others. No study explored the relationship between asthma and the individual’s personality. Further, research on psychosocial aspects of asthma was conducted in only 9 out of 22 countries comprising the Arab world.

The research literature advocates a multidisciplinary or team approach to management of bronchial asthma [71,72]. This approach which may involve such services as patient education, self-management, counseling and nutritional advice has been shown to improve quality of life and to reduce exacerbations and hospitalizations in patients with asthma [73,74]. The studies reviewed in the current review, however, revealed that this type of research is lacking in the Arab world. Accordingly, the need for further multidisciplinary research on the role of psychological factors in asthma management in Arab countries is clear. In addition, the program curricula in medical schools in the Arab world probably need to increase students’ awareness of psychological and behavioral aspects of illness, particularly chronic diseases having emotional components such as bronchial asthma. As Cuff and Vanselow [75] stated, measurable improvements in the health of individuals is unlikely to be achieved unless physicians are “equipped with the knowledge and skills from the behavioral and social sciences needed to recognize, understand, and effectively respond to patients as individuals, not just to their symptoms” (p. 3).

There is also a clear need for research addressing the potential effects of political and social turbulences on health status and care of individuals, including those with chronic illnesses such as bronchial asthma, in present-day Arab world. It is believed that “the psychological distress resulting from repetitive exposure to extreme forms of psychological trauma has put millions of civilians in the Middle East at high risk for trauma-related psychopathology” [76]. The “Arab Spring” which has touched many Arab countries recently may have significant consequences for population health. The violence and upheaval have resulted in significant political and economic challenges; difficult post-war conditions including influx of refugees throughout the region; under-employment and subsequent draining of financial, social, and health resources [77-79]. Another aspect that could affect health problems like asthma during “Arab Spring” is having high levels of psychological stress [76], a known serious trigger of asthma [80]. In a longitudinal cohort study [81] which investigated the role of psychological stress in the etiology of asthma, a strong association was found between stress and asthma incidence and hospitalization, use of asthma medication as well as with allergic rhinitis and atopic dermatitis in adults.

In addition, asthma is associated with environmental factors that can be increased during social unrest and civil wars, via means of fire, smoke, dust, and other toxic chemicals. Such events will not only have immediate impact, but can also have long-term effects. This has been illustrated in follow up studies [82,83] of the World Trade Center rescue and recovery workers which showed persistence of health problems such as asthma, sinusitis, gastro-esophageal reflux disease, depression, post-traumatic stress disorder, and panic attacks.

As mentioned previously, the majority of studies reviewed used self-report questionnaires to collect data from participants. It is well known that self-reports are subject to various sources of inaccuracies. Two major theoretical perspectives explain validity problems in some self-reported data [84]. The first is the cognitive perspective which attributes validity problems to inaccuracies arising from comprehension and recall. The second is the situational perspective which focuses on validity problems that arise from factors related to social desirability and interviewing conditions. Also, all studies reviewed used quantitative research methods in investigating the psychosocial aspects of bronchial asthma. However, research using qualitative methods such as in-depth interviews, case studies and participant observations [85] can yield important information by reflecting the experiences and stories of children and adolescents with bronchial asthma.

The present study has some limitations that need to be considered when interpreting the findings. First, this review was restricted to studies published in English and to studies published in referred journal articles. However, there may be studies published in Arabic or French (especially by researchers from Lebanon, Tunisia, Morocco, Algiers where French is widely spoken) or published as conference papers, master or doctoral theses, book chapters, etc. In addition, the studies originated from only 9 out of 22 Arab countries. These countries are quite diverse not only in terms of religion, history, economics, and politics but also in living standards and health care services. Finally, many studies reviewed involved non-probability or small samples. Therefore, caution should be exercised in making generalizations about psychosocial aspects of bronchial asthma in Arab countries.

## Conclusions

Research literature in Arab countries is lacking in quality studies investigating the psychosocial aspects of bronchial asthma. There have recently been some attempts to explore this research area; nonetheless, most studies carried out so far have investigated few psychological factors related to asthma. Further research involving randomized multicenter studies and using more rigorous research methods are needed to better understand the psychosocial aspects of bronchial asthma in the Arab world.

## Abbreviations

QoL: Quality of Life
UAE: United Arab Emirates
PAQLQ: Pediatric Asthma Quality of Life Questionnaire
PHC: Primary Health Care
CCAS: Chicago Community Asthma Survey
LWAQ: Living with Asthma Questionnaire
AGKQA: Asthma General Knowledge Questionnaire for Adults
WISC-R: Wechsler Intelligence scale for Children
WHO: World Health Organization
PSCL: Pediatric Symptom Checklist
